# Transactions of the Chicago Society of Physicians and Surgeons, December 6th, 1873

**Published:** 1874-01-01

**Authors:** Plym. S. Hayes


### Society Reports.

### TRANSACTIONS OF THE CHICAGO SOCIETY OF PHYSICIANS AND SURGEONS.

### MEETING OF DECEMBER 6TH, 1873.

*Reported by Plym. S. Hayes, M.D*

THE Society met in Parlor No. 1 of the Grand Pacific Hotel, the President, Dr. A. Fisher, in the chair.

The censors reported favorably on the application for membership of Drs. Charles Hayes, Simons, Tucker, Henrotin, jr., Hews, and Hamill, who were unanimously elected.

Dr. Wood read a paper on "Waxy Kidney," which he illustrated with a case from practice.

Dr. Jackson, chairman of the section of Obstetrics and Diseases of Women and Children, then read the report of the sub-section of Gynæcology, which consisted of extracts from the recent literature on the subject. The topics considered were: Cancer of the uterus treated by dried sulphate of zinc, ergot, and gastric juice; dysmenorrhœa treated by hysterotomy and mechanical dilitation, by means of bougies and laminaria digitata; use of fuming nitric acid in chronic inflammation of the uterus; intra-uterine medication; treatment of prolapsus by means of tannic acid; of procedentia by the actual cautery and pessaries; use of the intra-uterine stem in flexions; treatment of uterine fibrous tumors by hypodermic injections of ergotine, and of their removal by means of a steel wire; drainage tubes for the prevention of septicæmia, following ovariotomy; latent gonorrhœa in females; and dilitation of the female urethra.

After the reading of the report, the Doctor, by request, gave an account of two cases of fibroid tumors of the uterus, which were treated with hypodermic injections of ergotine. One of the cases had recovered, while the other was yet under treatment and improving. The solution used was one part of ergotine and three of water. In one case, the injection over the tumor was followed by abscesses. Subsequently, the region of the deltoid was selected, and the injection introduced deep into the muscular fibres, with as good result as when made over the tumor.

Dr. Owens described a method of treating the pedicle after ovariotomy, by means of an instrument which kept up a continuous traction on the wire ligature. He gave a description of the means employed in cleansing and disinfecting the abdominal cavity after ovariotomy, which consisted of a nasal douche that had inserted in the end of the tube a gum-elastic catheter, which was introduced into the abdominal cavity through the lower portion of the wound, the douche holding the liquid being at the same time held above the patient. When the douche was nearly emptied, it was held below the patient, thus forming a syphon of the tube, and emptying the abdomen.

Dr. Owens then presented a patient for examination by the members of the Society, of whose case he gave the following history: Mr. ——, aged thirty-three, contracted syphilis when in the army. Two years ago, the epiglottis, uvula, and part of the soft palate, were destroyed by syphilitic ulceration. The cicatrix contracting, left only a small orifice between the pharynx and the mouth. The patient could not talk above a whisper; and when eating or drinking, lies on his back, with head and shoulders somewhat elevated. He forms a bolus of his food, and, by means of the tongue, throws it into the throat, where it is grasped by the pharyngeal muscles, while the posterior portion of the tongue partially covers the larynx. He experiences more difficulty in swallowing liquids than solids; but rarely does anything enter the trachea.

Dr. Hyde submitted the following resolution, which was unanimously adopted:

WHEREAS, We have read the memorial relative to the medical corps of the army, addressed to the Senate and House of Representatives of the United States, which memorial was prepared in compliance with the unanimous resolution of the American Medical Association, adopted at its last meeting in St. Louis, Missouri; and, whereas, we heartily concur therein, therefore, be it

*Resolved*, That we, the Chicago Society of Physicians and Surgeons, respectfully invite the attention of our representatives in Congress to the memorial, and earnestly request their aid in securing the passage of the draft of a bill which accompanies it, entitled, "A bill to increase the efficiency of the medical department of the army."

Upon a motion of Dr. Jackson, the President appointed Drs. Jackson, Lyman and Davis a committee to prepare and submit a fee-bill to the Society.

Dr. Lyman moved that a committee be appointed to consult with the Chicago Medical Society in regard to the feasibility of having quarterly union meetings of the two societies. The motion was laid over until the next meeting.

Dr. Owens proposed the following resolution, which was carried:

*Resolved*, That a committee of two be appointed by the chair, to be known as the Committee on Clinical Reports, who shall cause to be presented to the Society monthly clinical reports from all medical institutions which may be found accessible.

The meeting then adjourned.

